# Supplementary material for: A Humanized Yeast Phenomic Model of Deoxycytidine Kinase to Predict Genetic Buffering of Nucleoside Analog Cytotoxicity
Source: Genes (Basel). 2019 Sep 30;10(10):770. doi: 10.3390/genes10100770 (PMC6826991; doi:10.3390/genes10100770)
Supplement: Supplementary file 1 [file genes-10-00770-s001.zip › Additional_Files/Additional_File7_GOTermHeatmaps.zip!/Additional_File7_GOTermHeatmaps/late_endosome_to_vacuole_transport.pdf]

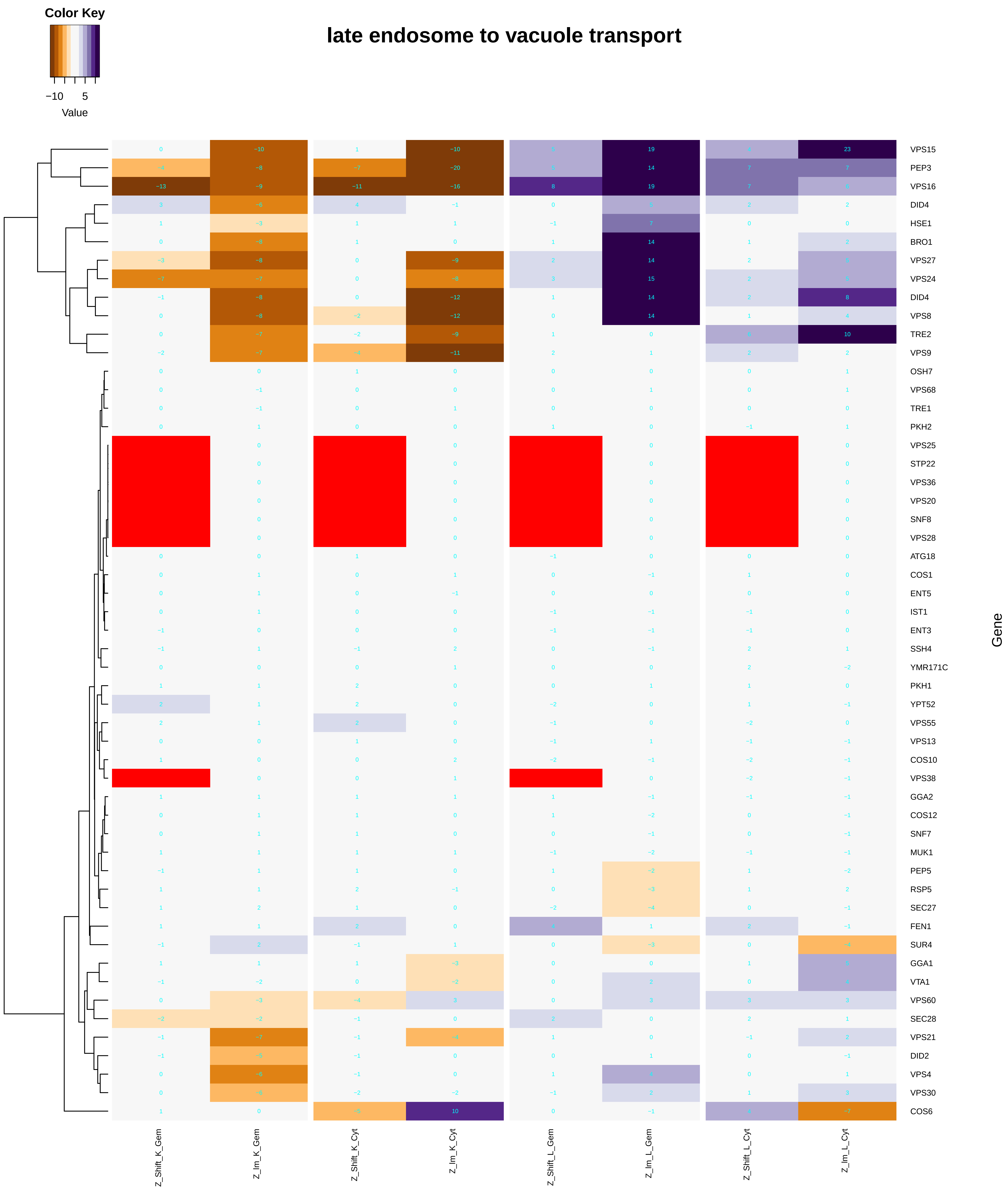
Color Key
−10
5
Value
late endosome to vacuole transport
VPS15
PEP3
VPS16
DID4
HSE1
BRO1
VPS27
VPS24
DID4
VPS8
TRE2
VPS9
OSH7
VPS68
TRE1
PKH2
VPS25
STP22
VPS36
VPS20
SNF8
VPS28
ATG18
COS1
ENT5
IST1
ENT3
SSH4
YMR171C
PKH1
YPT52
VPS55
VPS13
COS10
VPS38
GGA2
COS12
SNF7
MUK1
PEP5
RSP5
SEC27
FEN1
SUR4
GGA1
VTA1
VPS60
SEC28
VPS21
DID2
VPS4
VPS30
COS6
Gene
Z_Shift_K_Gem
Z_lm_K_Gem
Z_Shift_K_Cyt
Z_lm_K_Cyt
Z_Shift_L_Gem
Z_lm_L_Gem
Z_Shift_L_Cyt
Z_lm_L_Cyt

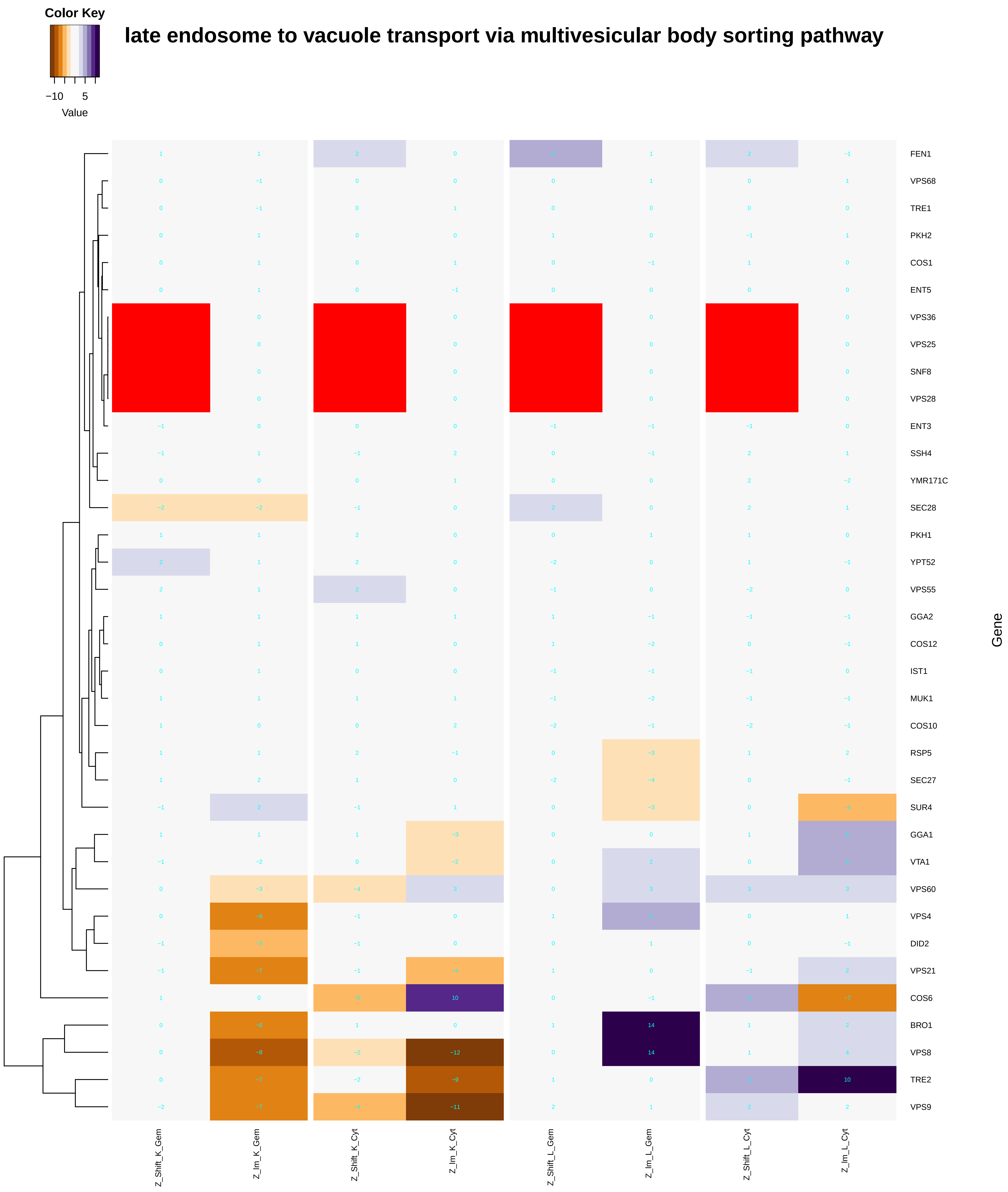
Color Key
−10
5
Value
late endosome to vacuole transport via multivesicular body sorting pathway
FEN1
VPS68
TRE1
PKH2
COS1
ENT5
VPS36
VPS25
SNF8
VPS28
ENT3
SSH4
YMR171C
SEC28
PKH1
YPT52
VPS55
GGA2
COS12
IST1
MUK1
COS10
RSP5
SEC27
SUR4
GGA1
VTA1
VPS60
VPS4
DID2
VPS21
COS6
BRO1
VPS8
TRE2
VPS9
Gene
Z_Shift_K_Gem
Z_Im_K_Gem
Z_Shift_K_Cyt
Z_Im_K_Cyt
Z_Shift_L_Gem
Z_Im_L_Gem
Z_Shift_L_Cyt
Z_Im_L_Cyt

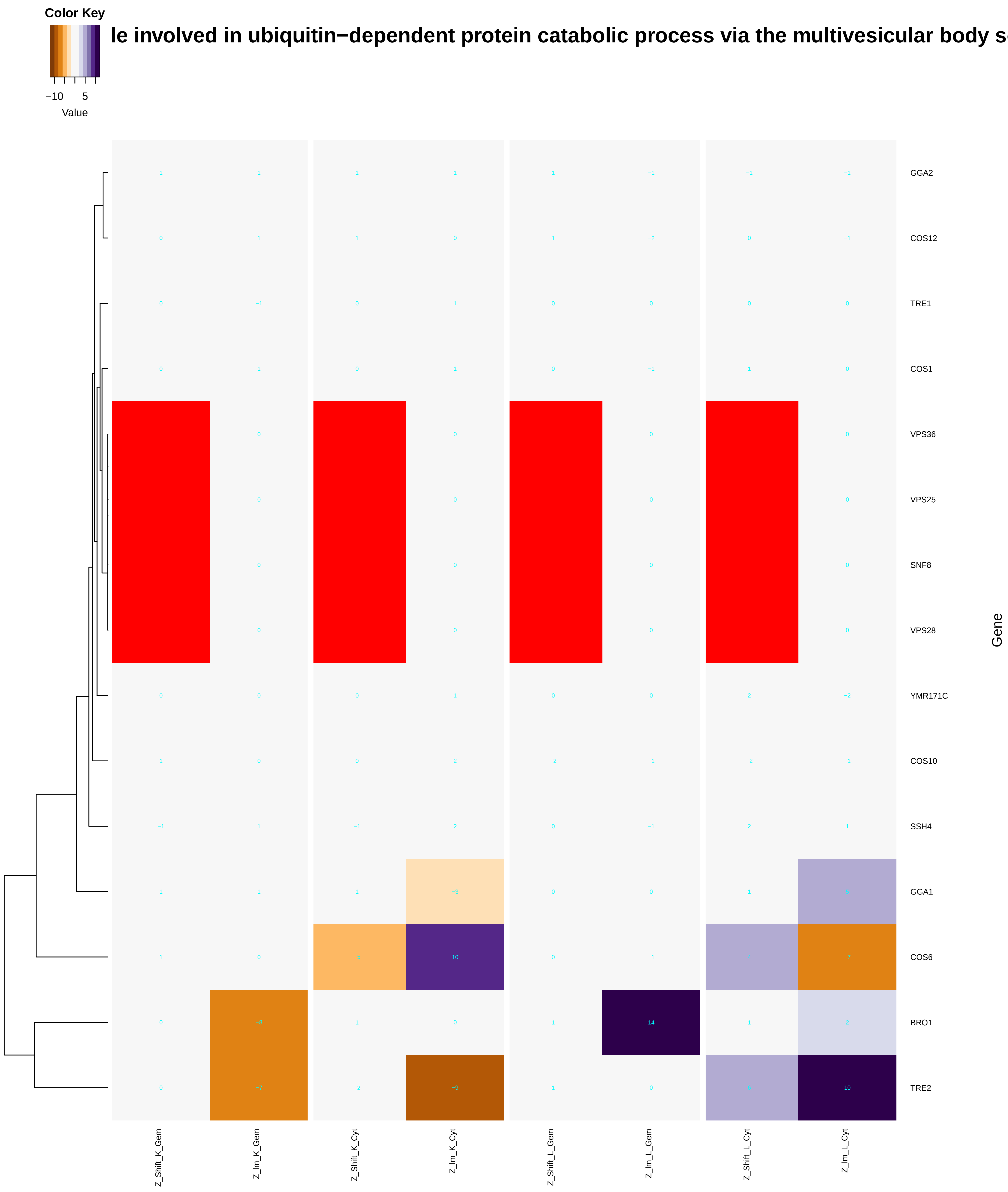
Color Key
le involved in ubiquitin−dependent protein catabolic process via the multivesicular body s
−10
5
Value
GGA2
COS12
TRE1
COS1
VPS36
VPS25
SNF8
VPS28
YMR171C
COS10
SSH4
GGA1
COS6
BRO1
TRE2
Gene
Z_Shift_K_Gem
Z_Im_K_Gem
Z_Shift_K_Cyt
Z_Im_K_Cyt
Z_Shift_L_Gem
Z_Im_L_Gem
Z_Shift_L_Cyt
Z_Im_L_Cyt
